# Supplementary material for: Unveiling the Bioactive Potential of the Invasive Jellyfish Phyllorhiza punctata Through Integrative Transcriptomic and Proteomic Analyses
Source: Biomolecules. 2025 Aug 4;15(8):1121. doi: 10.3390/biom15081121 (PMC12383608; doi:10.3390/biom15081121)
Supplement: Supplementary file 1 [file biomolecules-15-01121-s001.zip › Suplementary Material/FileS3/PEPFOLD4/XP_065054961_15200/clusters.html]

 PEP-FOLD clusters


Download archive of all models  here   

|  |  |  |
| --- | --- | --- |
| # | file | sOPEP |
| # cluster 1 - Size: 65 |  |  |
|  | XP\_065054961\_15200-model1 | -6.09249 |
|  | XP\_065054961\_15200-model1.1 | -6.08682 |
|  | XP\_065054961\_15200-model1.2 | -6.02893 |
|  | XP\_065054961\_15200-model1.3 | -6.00401 |
|  | XP\_065054961\_15200-model1.4 | -5.97961 |
|  | XP\_065054961\_15200-model1.5 | -5.9078 |
|  | XP\_065054961\_15200-model1.6 | -5.84747 |
|  | XP\_065054961\_15200-model1.7 | -5.83832 |
|  | XP\_065054961\_15200-model1.8 | -5.83266 |
|  | XP\_065054961\_15200-model1.9 | -5.80183 |
|  | XP\_065054961\_15200-model1.10 | -5.78045 |
|  | XP\_065054961\_15200-model1.11 | -5.74279 |
|  | XP\_065054961\_15200-model1.12 | -5.73254 |
|  | XP\_065054961\_15200-model1.13 | -5.72327 |
|  | XP\_065054961\_15200-model1.14 | -5.71517 |
|  | XP\_065054961\_15200-model1.15 | -5.68081 |
|  | XP\_065054961\_15200-model1.16 | -5.67843 |
|  | XP\_065054961\_15200-model1.17 | -5.65786 |
|  | XP\_065054961\_15200-model1.18 | -5.64479 |
|  | XP\_065054961\_15200-model1.19 | -5.63716 |
|  | XP\_065054961\_15200-model1.20 | -5.63435 |
|  | XP\_065054961\_15200-model1.21 | -5.55232 |
|  | XP\_065054961\_15200-model1.22 | -5.54337 |
|  | XP\_065054961\_15200-model1.23 | -5.50919 |
|  | XP\_065054961\_15200-model1.24 | -5.50795 |
|  | XP\_065054961\_15200-model1.25 | -5.50664 |
|  | XP\_065054961\_15200-model1.26 | -5.45945 |
|  | XP\_065054961\_15200-model1.27 | -5.45444 |
|  | XP\_065054961\_15200-model1.28 | -5.44731 |
|  | XP\_065054961\_15200-model1.29 | -5.39162 |
|  | XP\_065054961\_15200-model1.30 | -5.39085 |
|  | XP\_065054961\_15200-model1.31 | -5.38952 |
|  | XP\_065054961\_15200-model1.32 | -5.37819 |
|  | XP\_065054961\_15200-model1.33 | -5.34489 |
|  | XP\_065054961\_15200-model1.34 | -5.27112 |
|  | XP\_065054961\_15200-model1.35 | -5.25317 |
|  | XP\_065054961\_15200-model1.36 | -5.24546 |
|  | XP\_065054961\_15200-model1.37 | -5.2321 |
|  | XP\_065054961\_15200-model1.38 | -5.14791 |
|  | XP\_065054961\_15200-model1.39 | -5.09744 |
|  | XP\_065054961\_15200-model1.40 | -5.09527 |
|  | XP\_065054961\_15200-model1.41 | -5.08295 |
|  | XP\_065054961\_15200-model1.42 | -5.06941 |
|  | XP\_065054961\_15200-model1.43 | -5.06641 |
|  | XP\_065054961\_15200-model1.44 | -5.055 |
|  | XP\_065054961\_15200-model1.45 | -5.01468 |
|  | XP\_065054961\_15200-model1.46 | -4.81667 |
|  | XP\_065054961\_15200-model1.47 | -4.7976 |
|  | XP\_065054961\_15200-model1.48 | -4.73833 |
|  | XP\_065054961\_15200-model1.49 | -4.7297 |
|  | XP\_065054961\_15200-model1.50 | -4.67834 |
|  | XP\_065054961\_15200-model1.51 | -4.64621 |
|  | XP\_065054961\_15200-model1.52 | -4.64358 |
|  | XP\_065054961\_15200-model1.53 | -4.64221 |
|  | XP\_065054961\_15200-model1.54 | -4.58739 |
|  | XP\_065054961\_15200-model1.55 | -4.53448 |
|  | XP\_065054961\_15200-model1.56 | -4.51703 |
|  | XP\_065054961\_15200-model1.57 | -4.49523 |
|  | XP\_065054961\_15200-model1.58 | -4.48492 |
|  | XP\_065054961\_15200-model1.59 | -4.48331 |
|  | XP\_065054961\_15200-model1.60 | -4.42336 |
|  | XP\_065054961\_15200-model1.61 | -4.36537 |
|  | XP\_065054961\_15200-model1.62 | -4.3395 |
|  | XP\_065054961\_15200-model1.63 | -4.21785 |
|  | XP\_065054961\_15200-model1.64 | -4.21572 |
| # cluster 2 - Size: 1 |  |  |
|  | XP\_065054961\_15200-model2 | -6.06725 |
| # cluster 3 - Size: 3 |  |  |
|  | XP\_065054961\_15200-model3 | -5.66795 |
|  | XP\_065054961\_15200-model3.1 | -4.60185 |
|  | XP\_065054961\_15200-model3.2 | -4.19641 |
| # cluster 4 - Size: 15 |  |  |
|  | XP\_065054961\_15200-model4 | -5.33649 |
|  | XP\_065054961\_15200-model4.1 | -5.17373 |
|  | XP\_065054961\_15200-model4.2 | -5.17085 |
|  | XP\_065054961\_15200-model4.3 | -5.07222 |
|  | XP\_065054961\_15200-model4.4 | -5.05648 |
|  | XP\_065054961\_15200-model4.5 | -5.00145 |
|  | XP\_065054961\_15200-model4.6 | -4.94752 |
|  | XP\_065054961\_15200-model4.7 | -4.93827 |
|  | XP\_065054961\_15200-model4.8 | -4.90219 |
|  | XP\_065054961\_15200-model4.9 | -4.88468 |
|  | XP\_065054961\_15200-model4.10 | -4.83565 |
|  | XP\_065054961\_15200-model4.11 | -4.83201 |
|  | XP\_065054961\_15200-model4.12 | -4.82446 |
|  | XP\_065054961\_15200-model4.13 | -4.66252 |
|  | XP\_065054961\_15200-model4.14 | -4.481 |
| # cluster 5 - Size: 3 |  |  |
|  | XP\_065054961\_15200-model5 | -5.29027 |
|  | XP\_065054961\_15200-model5.1 | -5.24999 |
|  | XP\_065054961\_15200-model5.2 | -5.22088 |
| # cluster 6 - Size: 1 |  |  |
|  | XP\_065054961\_15200-model6 | -4.89866 |
| # cluster 7 - Size: 3 |  |  |
|  | XP\_065054961\_15200-model7 | -4.48314 |
|  | XP\_065054961\_15200-model7.1 | -4.33335 |
|  | XP\_065054961\_15200-model7.2 | -4.27139 |
| # cluster 8 - Size: 2 |  |  |
|  | XP\_065054961\_15200-model8 | -4.41901 |
|  | XP\_065054961\_15200-model8.1 | -4.3076 |
| # cluster 9 - Size: 2 |  |  |
|  | XP\_065054961\_15200-model9 | -4.30295 |
|  | XP\_065054961\_15200-model9.1 | -3.88356 |
| # cluster 10 - Size: 3 |  |  |
|  | XP\_065054961\_15200-model10 | -4.06858 |
|  | XP\_065054961\_15200-model10.1 | -4.01272 |
|  | XP\_065054961\_15200-model10.2 | -3.8637 |
| # cluster 11 - Size: 2 |  |  |
|  | XP\_065054961\_15200-model11 | -3.90164 |
|  | XP\_065054961\_15200-model11.1 | -3.65953 |
|  |  |  |
